# Mediastinal papillary thyroid carcinoma treated by video-assisted thoracic surgery – Case report

**DOI:** 10.1016/j.ijscr.2023.108140

**Published:** 2023-04-07

**Authors:** Teresa Vieira Caroço, Raquel Prata Saraiva, José Miguel Baião, Tiago Nogueira, Ana Luís Garcia, Carlos E. Costa Almeida

**Affiliations:** a General Surgery Department, Portuguese Oncology Institute of Coimbra, Av. Bissaya Barreto 98, 3000-075 Coimbra, Portugal; b General Surgery Department, Centro Hospitalar do Baixo Vouga, Av. Artur Ravara, 3814-501 Aveiro, Portugal; c Thoracic Surgery Department, Portuguese Oncology Institute of Coimbra, Av. Bissaya Barreto 98, 3000-075 Coimbra, Portugal; d Head and Neck Surgery Department, Portuguese Oncology Institute of Coimbra, Av. Bissaya Barreto 98, 3000-075 Coimbra, Portugal

**Keywords:** Ectopic thyroid, Mediastinal thyroid, Papillary carcinoma, VATS

## Abstract

**Introduction and importance:**

The mediastinal ectopic thyroid gland is rare and usually asymptomatic. Ectopic thyroid tissue has malignant potential, but ectopic thyroid cancers are extremely rare, particularly mediastinal thyroid cancer, with only five cases reported in the literature.

**Case presentation:**

A 73 years-old male patient diagnosed with multinodular goitre with two FLUS cytology was summited to an uneventful total thyroidectomy. Pathology revealed 8 synchronous papillary carcinomas in both thyroid lobes. Follow-up identified persistent elevation of thyroglobulin. A cervical ultrasound and cervical and thoracic CT scan were performed, identifying a mediastinal tumour of 6 × 3 cm. Resection was performed by video-assisted thoracic surgery (VATS). Pathology identified an ectopic mediastinal thyroid with a 4 mm papillary microcarcinoma. Recovery was uneventful and the patient is currently asymptomatic.

**Clinical discussion:**

There is no consensus on the best treatment strategy for mediastinal ectopic thyroid, but surgical resection is advised as being the only method allowing for a complete cure. Although both thoracotomy and sternotomy approaches have been usually used for mediastinal thyroid tumours resection, the thoracoscopic approach has been used with good results in recent years. Thoracoscopy has better visualization, less morbimortality, and faster recovery. Giant masses (>10 cm) are the only limitation for VATS.

**Conclusion:**

Ectopic mediastinal thyroid is extremely rare, and its malignant transformation is even rarer. There is no consensus on the best treatment strategy, but surgical resection of the mediastinal thyroid is advised. VATS is a safe and feasible minimally invasive technique with good outcomes.

## Highlights

• Mediastinal ectopic thyroid is a rare condition and usually asymptomatic.
• Malignant transformation is a possibility.
• Surgical resection is the best treatment for ectopic thyroid.
• VATS has a faster recovery, less morbidity, shorter length of stay, and better cosmesis.
• VATS is limited to mediastinal masses smaller than 10cm.

## 1 Introduction and importance

Ectopic thyroid is a rare entity resulting from abnormal embryological development of the thyroid [1], [2]. It can be found anywhere from the tongue to the diaphragm, and the most common location is the lingual, accounting for 90 % of cases [2], [3], [4]. Its prevalence is about 1 per 100,000 - 300,000 people, with 70–90 % of cases representing the only thyroid tissue found. There is a female preponderance, mainly in the Asian population [1], [2], [5]. Mediastinal ectopic thyroid is extremely rare, accounting for 1 % of all mediastinal tumours, usually located in the anterior mediastinum [1], [2], [5]. Cases of intrathoracic thyroid in the lungs and heart have been described [1]. Patients are usually asymptomatic and euthyroid, and because orthotopic tissue coexists, the diagnosis is usually incidental. However, dyspnoea, dry cough, and haemoptysis may be present. Superior vena cava syndrome or dysphagia may also occur but less frequently [1], [2], [6]. Ectopic thyroid tissue has the potential for malignancy and, although very uncommon, cases of carcinomas have been reported. As with orthotopic thyroid, the most common malignancy in ectopic thyroid is papillary carcinoma [1], [3], [6]. There are only five case reports of mediastinal ectopic thyroid carcinoma published in worldwide literature [4], [5], [6], [7], [8]. We present the sixth case report of a carcinoma arising in the ectopic mediastinal thyroid and the first treated by video-assisted thoracic surgery (VATS). This case is reported in accordance with SCARE guidelines [9].

## 2 Case presentation

A 73-year-old male patient resorted to the General Surgery consultation due to multinodular goitre, after seeing his General Practitioner because of cervical discomfort. A cervical ultrasound (US) identified a slightly enlarged thyroid gland with multiple nodules in both lobes, the bigger ones in the left lobe with 23 mm and 12 mm, without enlarged lymph nodes nor substernal extension. The patient was euthyroid with a TSH of 1.559 (0.55–4.78) and LT4 of 1.0 g/dL (0.89–1.76). A fine needle aspiration cytology (FNAC) of the bigger nodule was performed. Pathology concluded for a Bethesda III: follicular lesion of undetermined significance (FLUS). A second FNAC was performed 6 months later, with the same diagnosis. The patient was on antihypertensive medication and had no other relevant past medical history. The patient underwent an uneventful total thyroidectomy. During surgery, there were no features suggestive of malignancy, no extrathyroidal extension, no substernal extension, and no enlarged lymph nodes. The postoperative period was uneventful, and the patient was discharged home on the second day, taking levothyroxine 0.1 mg a day. Pathology revealed eight synchronous papillary carcinomas of the thyroid, all intraparenchymal, without perineural invasion nor vascular invasion, pT1b(m)NxMx. Of those, four were in the right lobe, ranging from 0.2 cm to 0.6 cm (microcarcinomas). The remaining four were in the left lobe, ranging from 0.6 cm to 1.8 cm.

**Fig. 1:**
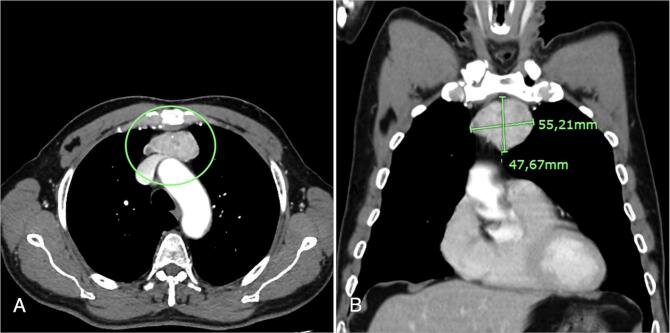
CT scan revealing a 55,21 × 47,67 mm tumour mass in the anterior and superior mediastinum (green circle); A – axial, B – coronal.

At 3 months of follow-up, a cervical US was performed revealing several unspecific nodules adjacent to the carotid artery. Thyroid function was difficult to control, ranging from subclinical hyperthyroidism to hypothyroidism, needing light adjustments in levothyroxine dosage. Additionally, serum thyroglobulin was elevated (31.71 ng/mL). Thus, a cervical and thoracic CT scan was performed, revealing a 60x30mm tumour mass in the anterior and superior mediastinum, hyperreflective and heterogenous, suggestive of tumoural recurrence or metastasis (Fig. 1). No lymph nodes were found. The perivascular nodules described in the US were not evident in the CT. Following a multidisciplinary discussion, surgical treatment was proposed, and accepted by the patient.

**Fig. 2:**
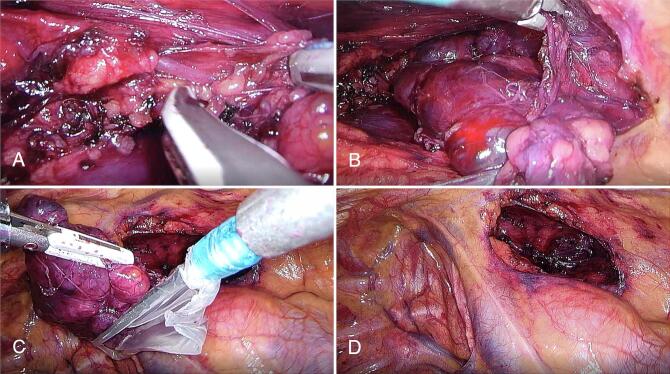
Resection of mediastinal mass by VATS; A – Ligation of intrathoracic vessels vascularizing the mediastinal mass with LigaSure Maryland Thoracic Sealer; B - blunt and sharp dissection of the mass with LigaSure Maryland Thoracic Sealer; C – retrieving the tumour with endobag; D – Final aspect of the mediastinum after removal of the tumour.

Mediastinal tumour resection was performed by video-assisted thoracic surgery (VATS). The patient was placed in left-side decubitus and three 10 mm trocars were placed on the right lateral thoracic wall (3rd, 5th, and 6th intercostal spaces). A 30° camera was used. The tumour was encapsulated in the anterior mediastinum with vascularization derived from intrathoracic vessels. There was no communication to the neck. Resection was performed using blunt and sharp dissection with LigaSure Maryland Thoracic Sealer (Medtronic) (Fig. 2). A drain was placed in the pleural cavity. The surgery was uneventful. The drain was removed the day after, and the patient was discharged home on the 2nd postoperative day free of symptoms.

**Fig. 3:**
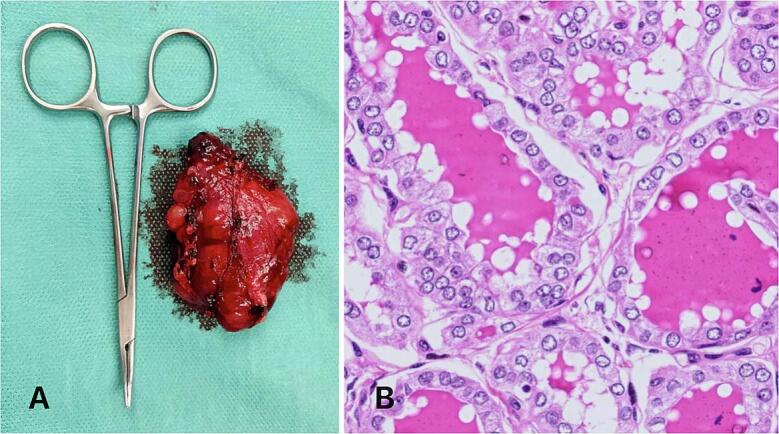
A – Final aspect of the mediastinal mass, size compared with Halsted mosquito haemostatic forceps; B – papillary microcarcinoma HE × 400: Microfollicles with cells with eosinophilic cytoplasm and enlarged nuclei, with clarified chromatin, longitudinal slits, and small nucleoli. There are no images of angioinvasion or vascular invasion.

Pathology concluded for an ectopic thyroid tissue of 55 × 45 × 25 mm, with multiple hyperplasic nodules and a 4 mm intraparenchymal papillary microcarcinoma, without perineural or vascular invasion (pT1aNxMx) (Fig. 3). After a second multidisciplinary discussion, no adjuvant treatment was delivered.

One month after surgery patient resumed normal activity without symptoms and with an excellent aesthetic result. Six months later the patient was doing well, with controlled thyroid function and serum thyroglobulin 0.33 ng/mL. No further imaging evaluation was performed so far.

## 3 Clinical discussion

Ectopic mediastinal thyroid only accounts for 1 % of mediastinal tumours. Differential diagnosis includes lymphomas, neurogenic tumours, mesenchymal and thymic tumours [1], [2], [3]. Although some reports of mediastinal ectopic thyroid can be found in worldwide literature, only five cases of malignancy have been reported. This is the first case reported of a mediastinal papillary microcarcinoma treated with VATS (Table 1).

**Table 1:** Mediastinal ectopic thyroid cancer cases described in the literature, ordered by date of publication.

Year	Authors	Sex/age	Diagnosis	Surgical approach	Pathology	Orthotopic thyroid
2007	Shah BC et al. [4]	F/45yo	CT and Tc-99m thyroid scan	Right lateral thoracotomy	Follicular variant of papillary carcinoma	Nodular goitre, no malignancy
2012	Piciu D et al. [5]	F/36yo	Whole-body scan with I-131 sodium iodide	N/A	Multifocal papillary carcinoma	Hashimoto Thyroiditis and a papillary microcarcinoma
2013	Shafiee S et al. [6]	F/39yo	CT	N/A	Papillary carcinoma	Normal histology
2017	Kita Y et al. [7]	F/71yo	CT	Median sternotomy	Follicular carcinoma with capsular invasion	N/A
2018	Karim F et al. [8]	F/55yo	CT	Mid-sternal body resection^a^	Follicular variant of papillary carcinoma	Normal histology
2021	Caroço TV et al.	M/72yo	CT	VATS	Papillary microcarcinoma	Multiple (8) papillary carcinoma

F – female; M – male, yo – years old; N/A – non available.

a Tumour invading the body of the sternum.

It is not uncommon for goitre to have a substernal extension into the superior mediastinum. It usually denotes the growth of the inferior thyroid tissue into de mediastinum with no separation from the cervical orthotopic gland [10]. On the contrary, ectopic thoracic thyroid has no direct connection with cervical thyroid tissue and has an independent arterial supply from the intrathoracic vessels, such as in the case reported [2], [4], [8], [10].

Once ectopic thyroid tissue can have a malignant transformation and is classically associated with an identical malignant transformation in the orthotopic thyroid, it can be challenging to differentiate ectopic thyroid carcinoma from metastasis [11]. In a literature review, three out of the five published case reports presented carcinoma in the mediastinal ectopic thyroid with normal orthotopic thyroid [4], [6], [8]. In our case, the malignant transformation of the mediastinal ectopic thyroid was synchronous to the papillary carcinomas found in the cervical thyroid. A different blood supply from the cervical gland and the absence of communication with the cervical gland prove its ectopy. Furthermore, to distinguish from a simple metastasis, the ectopic papillary microcarcinoma was found within thyroid tissue with nodular hyperplasia.

In case of suspicion of ectopic thyroid, scintigraphy with Tc-99m, I-131, or I-123, is the most valuable tool. It allows the identification of ectopic thyroid tissue as well as the presence of an orthotopic thyroid gland [1], [4], [6]. It is also sensitive and specific to differentiate from other mediastinal masses, such as thymoma or teratoma [2], [4]. Other diagnostic tools include colour doppler US, CT, or magnetic resonance imaging (MRI), which may help to identify the exact location of the mass and its relationships with adjacent structures, allowing for better preoperative planning [2], [8]. FNAC can be of good help, providing histological confirmation of ectopic thyroid tissue or even differentiating between benign or malignant lesions [6]. However, in most cases, the diagnosis is only made after the surgical removal of the tumour.

In the case presented, no FNAC of the mediastinal mass was performed since surgical approach was immediately decided facing the possibility of metastasis. Because of the high index of suspicion for thyroid cancer-related mediastinal mass, both endocrinologist and thoracic surgeon did not want to postpone resection. In that setting, no scintigraphy was performed either. Nevertheless, since thyroglobulin was elevated suggesting the persistence of thyroid tissue, scintigraphy should have been performed to accurately identify sites of remaining thyroid tissue. After mediastinal ectopic thyroid resection, thyroglobulin levels decreased below 1 ng/mL. No further imaging was performed. In case of persistent elevated serum thyroglobulin, scintigraphy would have been performed.

There is no consensus on the best treatment approach. As it is the only method allowing for a complete cure, most authors advocate complete surgical resection. In that setting, the size of the tumour, the age of the patient, airway obstruction, dysphagia, dysphonia, and compression of other adjacent structures, should all be considered in the decision-making process [1], [2], [3], [4], [8], [10]. Additionally, local complications, such as tumour ulceration, bleeding, cystic degeneration, and malignant transformation, should be considered [1].

Although thoracotomy or sternotomy have been usually required to remove mediastinal thyroid tumours in the past, the thoracoscopy approach has been recently reported with good results [4], [8]. Since the 2000s when VATS was initiated for thoracic sympathectomy, both its popularity and indications have increased, from atypical lung resections to standard lobectomies, or even mediastinal tumour resections [3], [12]. VATS has several advantages over open techniques: better visualization; less morbidity and mortality; less intra-operative blood loss; better final aesthetic result; faster recovery; shorter in-hospital length of stay [1], [3], [12]. The only limit seems to be giant mediastinal masses (>10 cm), which require classical thoracotomy or sternotomy, depending on the mass location [1], [3]. VATS is a safe and feasible technique. If complete resection of the mediastinal ectopic thyroid is achieved, the cure is accomplished [1], [3], [8]. In the case of remaining thyroid tissue, radioiodine therapy may be indicated [8].

We decided on a VATS approach considering all the advantages of this technique. Surgery was uneventful, with complete en bloc removal of the mediastinal mass. Recovery was fast as expected, and the patient was discharged home on the second postoperative day with pain easily controlled with a painkiller.

## 4 Conclusion

Ectopic mediastinal thyroid is extremely rare, and its malignant transformation is even rarer. There is no consensus on the best treatment strategy, but surgical resection of the mediastinal thyroid is advised. VATS is a safe and feasible minimally invasive technique with good outcomes.

## Consent

Written informed consent was obtained from the patient for publication of this case report and accompanying images. A copy of the written consent is available for review by the Editor-in-Chief of this journal on request.

## Ethical approval

Ethical approval was waived by the authors' institution.

## Funding

None.

## Guarantor

Teresa Vieira Caroço
Ana Luís Garcia
Carlos E. Costa Almeida.

## CRediT authorship contribution statement

Teresa Vieira Caroço – study concept, data collection and analysis, writing the paper, review
Raquel Prata Saraiva – data collection and analysis, writing the paper, review
José Miguel Baião – data collection and analysis, review
Tiago Nogueira – data collection, review
Ana Luís Garcia – writing the paper, review
Carlos E. Costa Almeida – writing the paper, review.

## Conflicts of interest

The authors declare no conflicts of interest.
